# Tocilizumab for massive refractory pleural effusion in an adolescent with systemic lupus erythematosus

**DOI:** 10.1186/s12969-021-00635-w

**Published:** 2021-09-16

**Authors:** Arianna De Matteis, Emanuela Sacco, Camilla Celani, Andrea Uva, Virginia Messia, Rebecca Nicolai, Manuela Pardeo, Fabrizio De Benedetti, Claudia Bracaglia

**Affiliations:** Division of Rheumatology, ERN RITA center, IRCCS Ospedale Pediatrico Bambino Gesù, Piazza Sant’Onofrio, 4-00165 Rome, Italy

**Keywords:** JSLE (Juvenile-onset Systemic Lupus Erythematosus) - Pleural effusion, IL-6, Tocilizumab

## Abstract

**Background:**

Pleural effusion in systemic lupus erythematous (SLE) is a common symptom, and recent studies demonstrated that IL-6 has a pivotal role in its pathogenesis.

**Case presentation:**

We report a case of a 15 years old Caucasian boy with a history of persistent pleural effusion without lung involvement or fever. Microbiological and neoplastic aetiologies were previously excluded. Based on the presence of pleuritis, malar rash, reduction of C3 and C4 levels and positivity of antinuclear antibody (ANA) and anti-double stranded DNA (dsDNA), the diagnosis of juvenile SLE (JSLE) was performed. Treatment with high dose of intravenous glucocorticoids and mycophenolate mofetil was started with partial improvement of pleural effusion. Based on this and on adults SLE cases with serositis previously reported, therapy with intravenous tocilizumab (800 mg every two weeks) was started with prompt recovery of pleural effusion.

**Conclusion:**

To the best of our knowledge, this is the first case of JSLE pleuritis successfully treated with tocilizumab.

## Introduction

Pleural effusion in systemic lupus erythematosus (SLE) occurs in 50 % of the patients. It is usually bilateral, small in size, asymptomatic and responsive to low dose glucocorticoid [1, 2]. We describe a patient with juvenile onset-SLE (JSLE) and massive refractory pleural effusion treated with tocilizumab (TCZ). Two cases of adult SLE patients with pleuritis successfully treated with TCZ have been reported [2, 3]. Interleukin-6 (IL-6) is a potential target in SLE given its role in anti-double stranded DNA (dsDNA) production and in the autocrine hyperactivity of B cells with spontaneous secretion of a large IL-6 amount and constitutive IL-6R expression [4–6].

## Case report

**Fig. 1:**
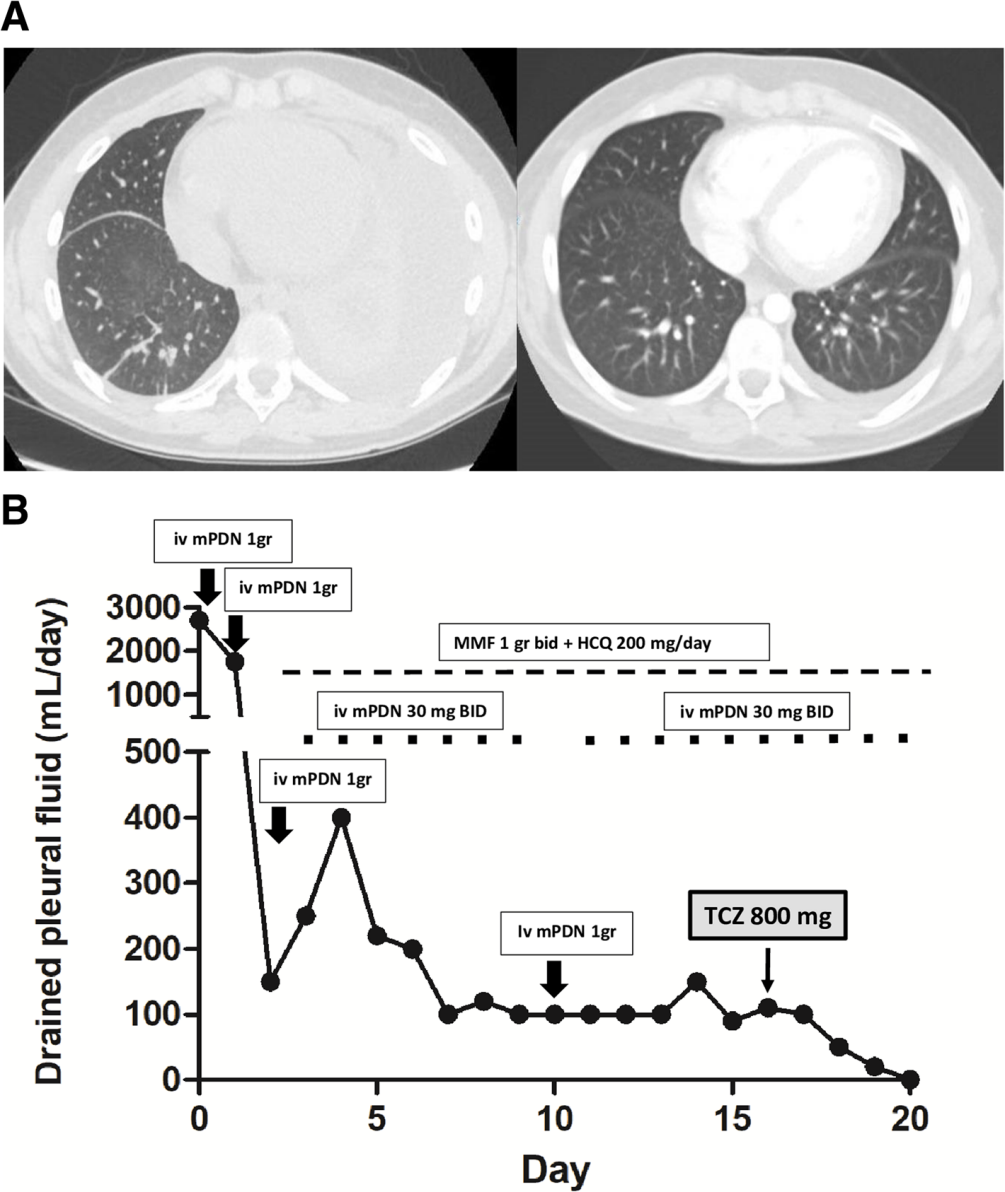
**a** Lung computed tomography (CT) before treatment with glucocorticoids and subsequent tocilizumab (left) and after (right) 9 days from the first dose of tocilizumab. **b** volume of drained pleural fluid and therapies administered. Arrows indicated 1gr mPDN iv pulses. BID: twice a day

A previously healthy 15-years-old Caucasian boy developed progressive dyspnoea and asthenia. At onset, laboratory features showed leukopenia (2.38 × 10^3^/uL), lymphopenia (0.89 × 10^3^/uL), hypergammaglobulinemia (22.31 g/l) and normal C-reactive protein (CRP) (< 0.5 mg/dl); antinuclear antibody (ANA) and anti-dsDNA were negative. Lung computed tomography (CT) demonstrates the presence of left pleural and pericardial effusion, without lung involvement and elevated hemidiaphragm (Fig. 1, panel A). He was treated with multiple antibiotics, with no response, and underwent four thoracenteses, with no microbial isolate. After two months, he was admitted to our division for the persistence of pleural effusion. He presented in poor general condition with tachypnoea, respiratory distress, requiring O_2_ supplementation, dullness over the left lung and mild malar rash. CRP, blood cell count, liver and kidney functional tests and urine analysis were normal. C3 and C4 levels were low. ANA, anti-dsDNA, anti-Sm and anti-U1RNP antibodies were present (Table 1). Type I interferon (IFN) signature was markedly elevated (score 76.4 median fold change). A massive left pleural effusion was was drained (2800 ml) and chest tube placed with marked improvement. Renal, cardiovascular and central nervous system involvement was excluded. A diagnosis of SLE was made according to the 2012 SLICC and 2019 EULAR/ACR criteria. Methylprednisolone (mPDN) pulses (1 gr/day, equal to 15 mg/kg/day, for 3 consecutive days) followed by mPDN 60 mg/day (equal to 0.8 mg/kg/day) were administered. Simultaneously mycophenolate mofetil (MMF) (1000 mg, equal to 600 mg/m2, twice a day) and hydroxychloroquine (HCQ) (200 mg/day, equal to 3 mg/kg) were also added. The daily volume of drained fluid mildly decreased (Fig. 1, panel B), and 10 days after one additional mPDN pulse was administered with no substantial effect. After 2 weeks, intravenous TCZ (800 mg, equal to 12 mg/kg, every two weeks) was started. Driven by the need for a rapid response before moving to other untargeted immunochemotherapies or invasive approaches (pleurodesis), we chose to use a high dose of TCZ to avoid the risk of under dosing. Immediately after the first dose, the daily volume of drained fluid decreased progressively allowing to remove the chest tube drainage on day + 4 after the first TCZ dose (Fig. 1, panel B). Nine days after the first TCZ dose, a chest CT documented no pleural effusion (Fig. 1, panel A). Prednisone (PDN) was progressively tapered without flares to 7,5 mg/day (0.09 mg/kg/day) at 9 months. After 6 months of treatment, blood tests improved (Table 1) and the infusion-interval of TCZ was extended to every 3 weeks without flares. After one year, the patient was in good clinical condition, still on TCZ (12 mg/kg every 3 weeks), oral PDN (5 mg/day, equal to 0.06 mg/kg/day), MMF (1 gr twice/day) and HCQ (200 mg/day). Pleural and pericardial effusions were not detected. Clinical inactive disease was maintained (SLEDAI 2), C3 and C4 improved but C3 of 0.75 g/l remained below the normal range (Table 1).

## Discussion and conclusion

Lupus pleuritis is caused by immune complex deposition, complement activation and direct binding of anti-dsDNA antibodies to mesothelium [7]. Several data suggest that IL-6 plays a critical role in B-cell hyperactivity and immunopathology of SLE and that it may have a direct role in mediating tissue damage [4]. A pilot study on 16 SLE patients and a trial on 15 SLE patients treated with TCZ showed a significant improvement in disease activity, and a significant reduction of levels of IgG and of anti-dsDNA [4, 5]. The trial also demonstrated that TCZ treatment significantly decreased the frequency of plasma cells and activated T cells, leading to a shift to a naïve B and T cells phenotype [4]. TCZ is not a standard treatment for SLE pleural effusion, with only two cases of adult patients reported [2, 3]. We chose TCZ because of its targeted mechanism, making it more selective, less immunosuppressive and with less potential short-term and long-term side effects compared to other conventional immunochemotherapy or pleurodesis.

Most likely, a combined effect of GCS, MMF and TCZ completely controlled pleural effusion, rapidly leading to inactive disease. However persistent mild low levels of C3 were observed. This may be due to neutralization of IL-6 activities, as reported in rheumatoid arthritis [8], rather than being a sign of disease activity, due to decreased liver production and not to consumption. Since a standardized protocol for the administration of TCZ in lupus patients is not available, the chosen regimen was based on the previous TCZ trials in SLE [4, 5]. Dosing regimen, timing of tapering and discontinuation of the drug are not defined and should be based on patient clinical condition.

**Table 1:** Laboratory parameters before treatment with tocilizumab (TCZ), after 6 months and after 12 months from starting TCZ (WBC = White blood cell count; N = Neutrophils; L = Lymphocyte count; Hb = Hemoglobin; PLT = platelet count; CRP = C reactive protein; SLEDAI = Systemic Lupus Erythematosus disease activity index); NA = not available.

	Baseline	72 h after TCZ	1 month after TCZ	6 months after TCZ	12 months after TCZ	Normal value
**WBC (x10**^**9**^**/L)**	4.0	9.91	5.4	4.2	4.4	4.0-13.5
** N (x10**^**9**^**/L)**	2.0	5.41	2.4	1.42	1.7	1.3–7.9
** L (x10**^**9**^**/L)**	1.5	3.73	2.5	2.2	2.25	1.0-6.5
**Hb (g/dL)**	11.2	12.5	13.7	12.5	12.9	13.0–16.0
**PLT (x10**^**9**^**/L)**	259	219	109	208	191	150–450
**CRP (mg/dL)**	0.6	< 0.03	0.03	0.03	0.03	< 0.5
**IgG (g/L)**	22.31		8.09	6.16	6.98	5.50–15.80
**C3 (g/L)**	0.36	0.57	0.71	0.67	0.75	0.90–1.80
**C4 (g/L)**	0.02	0.03	0.05	0.07	0.14	0.10–0.40
**Anti-dsDNA antibody score titer**	1:640	NA	1:20	1:10	1:10	< 1:10
**Anti-Sm antibody (U/mL)**	14480.0	NA	1589.0	496.0	428.0	< 5.0
**Anti-U1RNP IgG antibody (U/mL)**	370.0	NA	245	145.0	111.0	< 7.0
**SLEDAI**	14	6	2	2	2	≤ 4

## Data Availability

Not applicable.

## Abbreviations

ANA: antinuclear antibody
Anti ds-DNA: double stranded DNA
anti-Sm: anti-Smith
CRP: C-reactive protein
CT: computed tomography
HCQ: hydroxychloroquine
IL-6: interleukin-6
IL-6R: interleukin-6 receptor
JSLE: juvenile onset-SLE
MMF: mycophenolate mofetil
mPDN: methylprednisolone
PDN: prednisone
SLE: systemic lupus erythematous
SLEDAI: Systemic Lupus Erythematosus Disease Activity Index
TCZ: tocilizumab
